# Team-based primary health care for non-communicable diseases: complexities in South India

**DOI:** 10.1093/heapol/czaa121

**Published:** 2020-11-06

**Authors:** Dorothy Lall, Nora Engel, Narayanan Devadasan, Klasien Horstman, Bart Criel

**Affiliations:** 1 Institute of Public Health, 3009, II-A Main, 17th Cross, KR Rd, Siddanna Layout, Banashankari Stage II, Banashankari, Bengaluru, Karnataka, 560070 India; 2 Department of Health, Ethics & Society, CAPHRI Care and Public Health Research Institute, PO Box 616, 6200 MD Maastricht, The Netherlands; 3 Institute of Tropical Medicine, Nationalestraat 155, Antwerpen 2000, Belgium

**Keywords:** Teamwork, primary care, non-communicable diseases, implementation, quality improvement

## Abstract

Chronic non-communicable diseases (NCDs), such as diabetes and cardiovascular diseases, have reached epidemic proportions worldwide. Health systems, especially those in low- and middle-income countries, such as India, struggle to deliver quality chronic care. A reorganization of healthcare service delivery is needed to strengthen care for chronic conditions. In this study, we evaluated the implementation of a package of tailored interventions to reorganize care, which were identified following a detailed analysis of gaps in delivering quality NCD care at the primary care level in India. Interventions included a redesign of the workflow at primary care clinics, a redistribution of tasks, the introduction of patient information records and the involvement of community health workers in the follow-up of patients with NCDs. An experimental case study design was chosen to study the implementation of the quality improvement measures. Three public primary care facilities in rural South India were selected. Qualitative methods were used to gain an in-depth understanding of the implementation process and outcomes of implementation. Observations, field notes and semi-structured interviews with staff at these facilities (*n* = 15) were thematically analysed to identify contextual factors that influenced implementation. Only one of the primary health centres implemented all components of the intervention by the end of 9 months. The main barriers to implementation were hierarchical arrangements that inhibited team-based care, the amount of time required for counselling and staff transfers. Team cohesion, additional staff and staff motivation seem to have facilitated implementation. This quality improvement research highlights the importance of building relational leadership to enable team-based care at primary care clinics in India. Redesigned organization of care and task redistribution is important solutions to deliver quality chronic care. However, implementing these will require capacity building of local primary care teams.

KEY MESSAGES

- Implementing changes in the workflow, redistributing tasks to members of primary care teams, recording patient information and involving community health workers in the follow-up of patients in primary care settings in India is a challenge with respect to the local context.
- We found that the implementation of quality improvements was negatively impacted by the hierarchy within the team, inhibiting team-based care, whereas team cohesion and motivation from implementing the interventions facilitated the implementation process.
- We argue that there is a need to nurture participatory leadership at primary care facilities in India and similar settings to build an environment that overcomes hierarchies to facilitate team-based care.
- This study also highlights the need for more research regarding organizational behaviour at primary care facilities in India to strengthen primary health care.

## Introduction

In the last few decades, the burden of chronic non-communicable diseases (NCDs), such as diabetes and cardiovascular diseases, has increased, especially in low- and middle-income countries (LMICs), including India (Dandona *et al.*, 2017). India is heralded as the diabetes capital of the world, with an estimated 72 million persons living with diabetes in 2017 (International Diabetes Federation, 2017). Hypertension, a leading risk factor for cardiovascular disease, has also steadily increased. In 2017, there were an estimated 207 million persons living with increased blood pressure in India (Gupta *et al.*, 2018).

Traditionally designed to deliver care for acute diseases, health systems in India are struggling to provide care for chronic conditions (Samb *et al.*, 2010; Gabert, 2017). Despite a national programme launched by the government of India in 2009 for the control of NCDs, focusing on diabetes, stroke, cancer and cardiovascular diseases (Ministry of Health & Family Welfare and Government of India, 2010), the outcomes of care for these conditions are abysmally poor (Mohan *et al.*, 2014; Unnikrishnan *et al.*, 2014). Primary care is best suited for managing chronic NCDs (Rothman and Wagner, 2003; World Health Organization, 2008), but the services specified in the national programme are limited to screening, continuation of medication and providing health-promoting messages. Strengthening primary care to deliver continuous, comprehensive and coordinated care for persons with chronic conditions is necessary (Das *et al.*, 2015; Sinha and Pati, 2017). Any improvements in the design of services require evidence about the system changes needed to produce better care (Wagner *et al.*, 2001).

Quality improvement (QI) research studies the design, development and evaluation of interventions to provide evidence for relevant redesign of health systems (Eccles *et al.*, 2003; Peters *et al.*, 2013). There is a paucity of literature from India with regard to such QI initiatives, especially at the primary care level, that could inform the design and guide the implementation of interventions meant to improve care for chronic conditions.

We conducted QI research to improve care for chronic NCDs, specifically diabetes and hypertension, at public primary health centres (PHCs) in a rural district in South India. The interventions to improve quality of services for diabetes and hypertension care were developed to address specific gaps identified through a situational analysis in the same setting as reported previously (Lall *et al.*, 2019).

In this paper, we present the implementation process, including the development of interventions. We critically analyse the implementation process using implementation and QI frameworks to identify contextual factors that may have resulted in the differential uptake of interventions at the different PHCs. The insights we gained are important to consider while redesigning the delivery of services and may have implications on the implementation of the current national programme for NCDs.

### Theoretical background

The quality of care delivered through the health infrastructure is of great concern to health care providers and patients. The National Academy of Medicine (previously Institute of Medicine) landmark report on crossing the quality chasm catalysed discussions on delivering and measuring quality in health care organizations (Institute of Medicine, 2001). Arguably, improving the quality of services is an everyday task and an obligation of service providers (Hirschhorn *et al.*, 2018), but systematically studying the effect of changes to the delivery process on outcomes of care can inform choices that providers make. Thus, QI research is characterized as a type of implementation research (Peters *et al.*, 2013). Interventions in QI research are usually complex, and their success often depends on how the approach is tailored to address a problem in a given context (Ramaswamy *et al.*, 2018). Contextual influences include all factors other than the intervention that influence implementation and outcomes of QI (Damschroder *et al.*, 2009; Wells *et al.*, 2012). A systematic review of contextual factors in 47 QI studies revealed that organizational characteristics of the health facilities (e.g. size, ownership), leadership of the QI and management teams, organizational culture, number of years involved in QI, data infrastructure and information systems and resources available are associated with implementation outcomes. Several theoretical frameworks have attempted to categorize these contextual factors and are useful, as they allow a systematic assessment of context in a wide range of settings (Greenhalgh and MacFarlane, 2005; Johnson and May, 2015; May et al., 2016). In this analysis, we draw on two such theoretical frameworks, the Consolidated Framework for Implementation Research (CFIR) (Damschroder *et al.*, 2009) and the Model for Understanding Success in Quality (MUSIQ) (Kaplan *et al.*, 2012), to analyse factors that may have influenced implementation in our setting and context.

The CFIR is a meta-theoretical framework that identifies five major domains that impact implementation: FEFFthe intervention, inner and outer settings, the individuals involved and the process by which implementation is accomplished. The intervention is conceptualized as having core components and elements that can and should be adapted to the setting. The inner settings relate to the economic, political and social context within which the organization resides, and the outer settings to the structural, political and cultural contexts through which the implementation process proceeds. The individual is viewed as the carrier of cultural, organizational, professional and individual mindsets, norms, interests and affiliations with predictable or unpredictable consequences for implementation. Finally, the implementation process itself involves an active change process aiming to achieve use of the interventions as designed (Damschroder *et al.*, 2009). This framework provides useful categorization and definitions of the factors affecting implementation that have been reported in implementation literature.

The second framework was more specific to understanding the context of QI in health care. The MUSIQ identifies external environment, organization (including QI leadership), microsystem (including data infrastructure) and QI team (including physician involvement) as broad categories of factors that influence implementation. Though the CFIR also mentions these categories, the MUSIQ provides a model for understanding how these categories interact to affect implementation (Kaplan *et al.*, 2012).

## Methods

To study the process of implementation and identify influential factors, we chose the case experimental design (Peters *et al.*, 2013; Toulany *et al.*, 2013) including both observation and the implementation of interventions to improve quality. The interventions were not static, but changed as they were adapted in the course of implementation relevant to the context of the individual setting.

### Setting

The study was conducted at three publicly funded primary care facilities in Kolar, a rural district in Karnataka state in South India with a population of 1 536 401 (Office of the Registrar General and Census Commissioner India, 2011). In India, health care is ordered in a three-tiered system of primary, secondary and tertiary care provided by both the public and private sectors. >70% of care for chronic conditions is provided by the private sector, and the public sector mainly provides services for those who are unable to afford care elsewhere (Balarajan *et al.*, 2011).

Primary care is delivered by the public sector at PHCs, which are the first point of contact with a medical doctor, and through a network of sub-centres where an auxiliary nurse midwife and community health workers [accredited social health activists (ASHAs)] are available for a population of 5000 and 1000, respectively.

Kolar district has 60 PHCs that, in principle, cater to a maximum population of 30 000 persons in a defined catchment area of surrounding villages (Directorate General of Health Services, 2012). The usual team at a PHC includes the medical doctor, two staff nurses, a lab technician and a pharmacist. Services provided at the PHC are largely structured through ‘vertical’ disease control programmes to fight-specific diseases or disorders, e.g. tuberculosis, blindness and malaria. The National Program for Control of Diabetes, Cancers and Stroke (NPCDCS) is the NCD programme specifying a package of services be delivered at the PHC that includes screening for diabetes and hypertension, referral to a secondary level hospital for diagnosis, and continuation of medication for those diagnosed (Ministry of Health & Family Welfare, 2016). Kolar district has been implementing this programme since 2009. The doctors in charge of PHCs are responsible for the centre’s performance.

### Selection of PHCs

As the QI initiative sought to improve the quality of care for diabetes and hypertension, we considered the average number of patients with diabetes and hypertension being treated at the PHC and the presence of basic infrastructure, including medicines and health professional staff, to create a list of 25 PHCs that were eligible as ‘cases’ for the study. The list was then discussed with the district health officer (DHO) and 10 of the PHCs shortlisted. Finally, three PHCs were selected from this list based on the willingness of the medical doctor to participate.

All three PHCs had a doctor, two nurses, a lab technician and a pharmacist at the time of inclusion in the study (Table 1). PHC 2 also had a care coordinator (CC) with a non-health background. The CC was appointed to coordinate another pilot project to create digital records of persons in the PHC catchment area. PHCs 1 and 2 were similar to respect to the average number of patients, and the average number of persons with diabetes and hypertension receiving treatment. PHC 3 had a larger catchment area population but relatively fewer patients with diabetes and hypertension under treatment at the PHC.

**Table 1:** Description of the selected PHCs

Characteristic	PHC 1	PHC 2	PHC 3
Location from the closest town area	Close	Remote	Close
Team at PHC	MO, 2 nurses, lab technician, pharmacist	MO, 2 nurses, lab technician, pharmacist, CC	MO, 2 nurses, lab technician, pharmacist
Population in the catchment area	16 000	12 000	31 000
Average daily number of patients seen in OPD	120	100	60
Average monthly number of persons with DM or HTN	20	40	18

CC, care coordinator; DM, diabetes mellitus; HTN, hypertension; MO, medical officer; OPD, outpatient department.

### Intervention development

**Figure 1:**
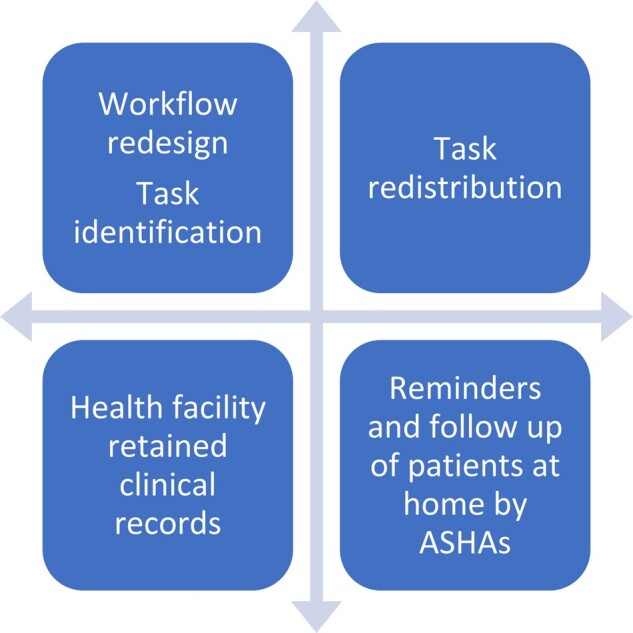
Elements of the intervention package

We developed the interventions based on the results of an analysis of the quality of service delivery for diabetes and hypertension at PHCs in Kolar district that we reported elsewhere (Lall *et al.*, 2019). Doctor-centred care processes, lack of information to maintain continuity of care, fragmented care processes, poor support for self-management and decision-making that was not evidence-based or patient-centred were identified as factors impacting the quality of care for NCDs (Lall *et al.*, 2019). We presented these findings to the DHO and programme managers of the NPCDCS in Kolar district to identify possible interventions to address the gaps. The interventions were then discussed with other co-authors who had relevant experience in improving quality of care in similar settings. A package of interventions (Figure 1) was arrived at and further refined based on the recommendations of the WHO, such as the package of essential NCD interventions (WHO, 2010) and the innovative care for chronic conditions framework (World Health Organization, 2001). These interventions were also supported by evidence of effectiveness in the literature regarding QI for NCDs at the primary care level. The proposed interventions were a redesign of the workflow for patients with diabetes and hypertension and identification of tasks to be completed in the care of these patients (Knox and Branch, 2015; Panattoni *et al.*, 2017); (Unertl *et al.*, 2009); allocation and redistribution of tasks among the staff at the PHC (Joshi *et al.*, 2014; Gyamfi *et al.*, 2017); record patient information at the health facility (Wagner *et al.*, 2001; Unertl *et al.*, 2009) and involve ASHAs to follow-up with patients in the community (Gilmore and McAuliffe, 2013; Jacobs *et al.*, 2015).

The interventions were then locally adapted and co-designed with the health care teams at each of the three PHCs during the course of the study. Co-designing practically entailed iterative discussions with the staff to determine the exact details of the intervention bundle. An average of four such discussions or planning meetings was conducted at each PHC with the staff before we began implementing the interventions. This participatory approach was inspired by the action research methodology (Whyte, 1991) relevant to QI initiatives, and is known to increase ownership of such initiatives (Wolstenholme *et al.*, 2017). Our role as a research team was to facilitate the discussions. In response to practical problems that the teams faced, minor changes were made to the interventions during implementation. Consequently, the interventions were slightly different at each of the PHCs, even if broadly similar.

The study received ethical approval from the ethics committees of the Institute of Tropical Medicine, Antwerp (ref 1186/17), the University of Antwerp (ref 17/47/527) and the Institute of Public Health, Bengaluru (ref IEC-FR/02/2017). We also obtained permission from the State Ministry of Health and the Kolar District Health Office to conduct this research.

### Capacity building

The primary author and a research associate conducted training for the staff regarding NCDs, risk factors and their control relevant to the roles of counsellor, lab technician, pharmacist and physician when the staff expressed a lack of skills, such as counselling, that were necessary to fulfil the tasks they volunteered to complete. The Indian guidelines for the standard treatment of diabetes and hypertension (National Centre for Disease Control, MOHFW and GOI, 2017) and the NPCDCS operational guidelines (Ministry of Health & Family Welfare and Government of India, 2010) were used as reference documents.

No format was available to record patient information at the PHCs. Therefore, the primary author and a research associate consulted with the staff to create a format (Supplementary 1). The development of the tool required four iterations to balance the main concern of the staff regarding the time it would take to record the information and identify the necessary information required for treatment decisions.

### Data collection

The primary author and research associate made an average of 14 visits to each PHC to monitor the interventions during the 9 months of implementation. At each visit, observations were conducted and extensive field notes taken, specifically including information related to the care for NCDs. We also conducted semi-structured, in-depth interviews with the teams at the three PHCs (*n* = 15) after 9 months (January–September 2018) of the implementation (Table 2). In addition to the doctor, nurse, lab technician and pharmacist, at PHC 2 we also interviewed the CC. At PHC 3, we were not able to interview the doctor or the pharmacist, as they had been posted elsewhere.

**Table 2:** Team at each PHC

PHC		Interviewee	Label	Age range (years)	Number of years at PHC
1		Nurse 1	N1a	30–40	5–10
		Nurse 2	N1b	20–30	<1
		Medical officer	M1	20–30	1–5
		Lab technician	L1	40–50	15–20
		Pharmacist	P1	50–60	10–15
2		Nurse 1	N2a	20–30	1–5
		Nurse 2	N2b	30–40	1–5
		Medical doctor	M2	20–30	1–5
		Lab technician	L2	30–40	10–15
		Pharmacist	P2	40–50	10–15
		CC	CC2	20–30	<1
3		Nurse 1	N3a	30–40	5–10
		Nurse 2	N3b	20–30	<1
		Lab technician	L3	30–40	1–5
		Medical doctor	-	30–40	1–5
		Pharmacist	-	30–40	5–10

The objective of the interviews was to understand how the staff viewed the interventions and their implementation. The interviews included questions to explore the working environment, their ability to make changes, the challenges they faced and their motivation to implement the interventions. The interview guides were pilot-tested and refined prior to the interviews (Supplementary 2). All of the interviews were conducted at the PHC at a time convenient for the respondents that did not interfere with their daily work schedule. Each of the interviews was conducted with due attention to privacy, and consent was obtained individually before the interviews. Interviews lasted an average of 30 min. During the interviews, interpretations were checked with the participants (member validation) to improve the internal validity of the data.

### Positionality of researchers

Although the primary author and research associate were considered external to the PHC team, in the initial months they were invited on 4–5 occasions to participate in some of the tasks, such as recording patient information and directing patients to follow the new workflow. In the initial few months, we also had to remind the team at each PHC to complete the records on 2–3 occasions. However, we maintained an outsider position throughout the research and were reflexive, especially as our backgrounds as health care professionals could have influenced the interpretations. The outsider position enabled an objective as possible assessment of the implementation process.

### Data analysis

Data from the observations, interviews and field notes were uploaded to NVivo (QSR International Pty Ltd., version 11, 2015). Coding was done by the primary author using codes identified a priori from relevant constructs of the CFIR and MUSIQ. New codes were assigned to data that did not fit the a priori codes. Codes, such as culture of the PHC, implementation climate, attitude to intervention and motivation of staff, were used to categorize the data. Both inductive and deductive approaches were used in the analysis of text. Repetitive reading of the data and comparisons with the constructs in the frameworks were performed to refine the codes. Coding was discussed with the other authors to develop a coding tree.

We analysed the data to understand *how much of what was planned* was implemented at each of the PHCs over the 9 months. A thematic analysis was then conducted to identify the themes of contextual factors that may have impacted the implementation process. The differences in context at the three PHCs were compared with identify possible explanations for the findings. Participant observation data were used independently to triangulate the data from the interviews. We found no inconsistencies or contradictions in the data.

## Results

**Figure 2:**
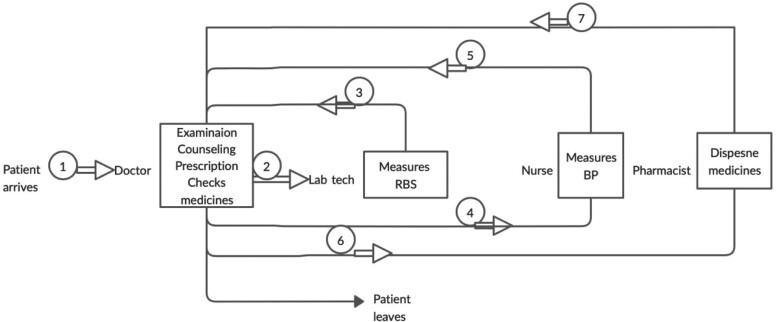
Pre-intervention workflow and task distribution at the PHCs. BP, blood pressure; Lab Tech, laboratory technician; RBS, random blood sugar

The workflows at the three PHCs were similar before the start of the intervention (Figure 2). Most tasks, such as examination, counselling and prescribing, were done by the doctor. Notably, no foot examination was done and, although the doctor was counselling patients, it was inconsistent and limited due to time constraints.

### PHC 1

We conducted four planning meetings at this PHC during the first 3 months of the study (January–March 2018). The doctor decided which members of the team would attend; each meeting was attended by two members in addition to the doctor, and at no meeting were all four members present. The discussions were interactive, but the allocation of tasks and decisions regarding changes were made by the doctor.

The first QI measure we discussed was the flow of patients and identification of tasks. Members of the team charted the prevalent flow of a patient diagnosed with an NCD and suggested changes from a patient’s perspective (Table 3). A new task identified was measurement of fasting blood sugar, but this was met with resistance by the doctor who felt that it would alter the routine of the PHC and be unacceptable to patients.

**Table 3:** Patient flow and tasks at PHC 1 during the course of implementation

PHC 1	
Intervention	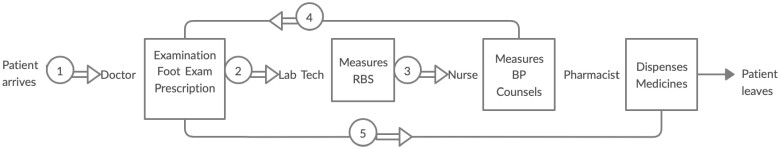
6 months	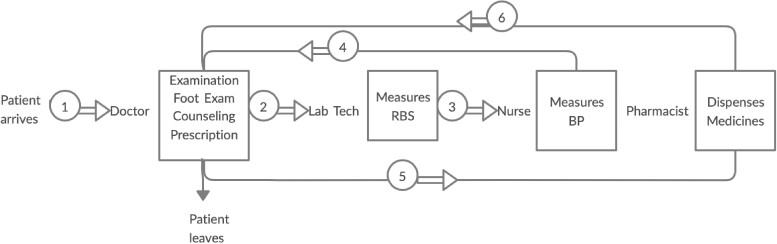
9 months	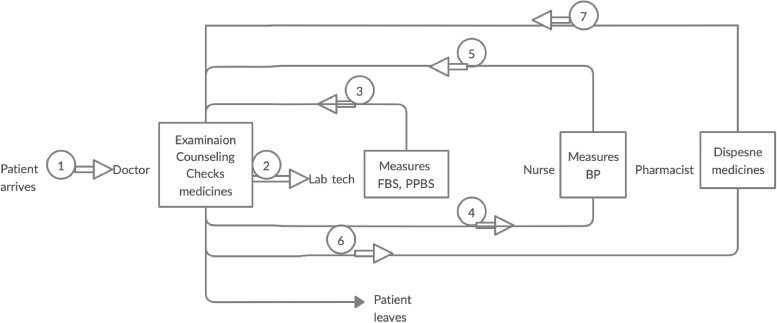

BP, blood pressure; FBS, fasting blood sugar; Lab Tech, laboratory technician; PPBS, post prandial blood sugar.

*No … it’s [testing fasting blood glucose before 9AM] not a tradition here … even if you call the patients also … they will come at 11:30 or so …* (D1).

The workflow was implemented as planned for 2 months but changed after 6 months, and at the end of 9 months the workflow was almost similar to the pre-intervention period (Table 3). The doctor felt this was because of the large number of patients and the inability of patients to adjust to the new workflow.

*Patient flow, usually it will look nice for only 2 to 3 months, again the patients will follow what they are used to, they will come to the doctor they will see, they will take medicine … the [new] flow will be difficult when the crowd is more …* (D1).

The second QI included the distribution of tasks among team members, such as measurement of blood pressure and counselling by the nurse, foot examination by the doctor, measurement of blood glucose by the lab technician, and dispensation of medicine by the pharmacist. The third QI, recording patient information, was to be done by all members relevant to the task they complete. The implementation of the second and third improvements followed a pattern similar to the workflow and, by 9 months, most of the tasks and recording were being conducted by the doctor (Table 3). Consequently, even though >100 patients were seen by the end of 9 months, only 52 records had been initiated. A lack of time was identified as the reason for not implementing the interventions.

*The patients are large in number, nobody has time to sit with each of them and talk to them* (N1a).

*We thought of … giving a specific work for a specific people but it didn’t work really … because staff nurse is busy in pricking [injections] … and the pharmacist is busy, only thing is a doctor is free in this* (D1).

We observed that the doctor did not take the initiative to involve members of the team:

*Since the doctor sees the patient and prescribes the tablets, he will only maintain the record, rather he explaining us how to do, he is only writing it* (LT1).

The fourth QI to involve ASHAs in the follow-up was initiated in one meeting where they were sensitized to their role in follow-up and to support lifestyle modification. This was attended by most of the ASHAs (15 of 20). The doctor also conducted one meeting, where he used the clinical record to identify patients in each ASHA’s area. However, there was no follow-up meeting to continue this element of the intervention.

### PHC 2

Four planning meetings were conducted at this PHC, and each was attended by all of the staff. The members of the team waited for each other to finish their work, and these meetings were conducted during the lunch break. Even though the doctor assumed the lead role and gave instructions, there was room for the staff to express their opinions and make suggestions with respect to specific QIs. Discussions began with charting the prevalent workflow and tasks, followed by redesigning and redistributing tasks from a patient’s perspective.

The workflow design, task distribution and recording of patient information were implemented at the end of 9 months (Table 4).

**Table 4:** Patient flow and tasks at PHC 2 during the course of implementation

PHC 2	
Intervention	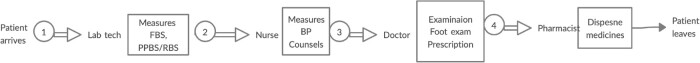
6 months	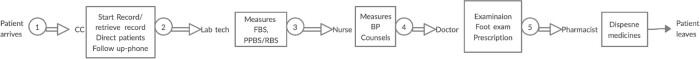
9 months	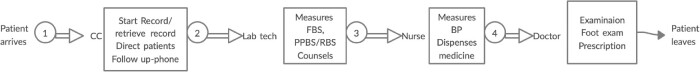

BP, blood pressure; CC, care coordinator; FBS, fasting blood sugar; Lab Tech; lab technician; PPBS, post prandial blood sugar.

A total of 210 patients had clinical information recorded and used for treatment decisions. Coincidentally, 1 month after the start of the implementation, a new staff member, a CC, was posted at the PHC as part of another district health project. The CC took on the role of coordinating the workflow and guiding patients. The CC became the holder of patient records and was responsible for registering new patients and identifying cards for repeat patients. This was not a role the team had envisioned at the start, but it enabled implementation of the workflow:

*First, they used to go to the doctor, and then come to me get sugar levels checked, and then go to the doctor, and if doctor says, they come back and get their BP checked. Now it is not like that, first they get their cards from CC and get their tests done then visit the doctor* (LT2).

Changes to the task distribution were made at monthly meetings, where staff had an opportunity to discuss the challenges they faced. The task of dispensing drugs was initially the responsibility of the pharmacist, but during the course of implementation, it was taken on by the nurse for efficiency (Table 4). Similarly, counselling was initially taken up by the nurse, but as the lab technician had more time and was interested in counselling, she volunteered to do this task. The counselling was done when patients had their blood sugar tested, and each session lasted 5–20 min. Counselling included advice regarding lifestyle modifications and often became an opportunity for patients to discuss challenges in their lives, including family circumstances.

*When we counsel about walking, food habits, to avoid drinks and betel nut leaves, they sit and listen. By doing all these, they can avoid problems, so they sit and listen* (LT2).

Fasting blood glucose testing was a challenge and could not be implemented initially because it required the lab technician to be available earlier than the regular time. However, the night shift nurse volunteered to take on this task and, by 9 months, this it was implemented. However, ASHAs were not involved because the CC took on the task of making phone calls and reminding patients about follow-up. 

### PHC 3

At this PHC, we conducted five planning meetings attended by all of the staff. The doctor at this PHC assumed the lead responsibility, but the discussions were interactive and the staff participated in decision-making regarding the workflow and responsibilities. At 6 months, most of the QI interventions had been implemented as planned by the team (Table 5).

**Table 5:** Patient flow and tasks at PHC 3 during the course of implementation

PHC 3	
Intervention	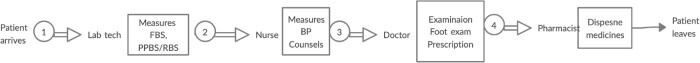
6 months	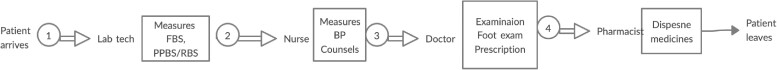
9 months	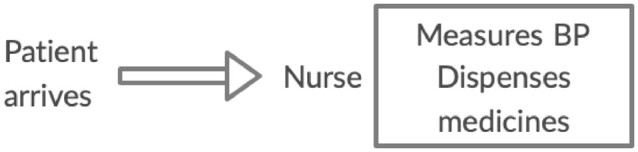

BP, blood pressure; FBS, fasting blood sugar; Lab Tech, laboratory technician; PPBS, post prandial blood sugar; RBS, random blood sugar.

The nurse expressed challenges, especially with the counselling, as she could not cope with the time it required. On two occasions, the time she took to counsel led to the patients waiting outside becoming restless, and they demanded to be seen faster. This was discussed at a follow-up meeting and the lab technician volunteered to do this task when she tested patients’ blood sugar levels.

*We can do the counselling, Madam, but we do not have time, we cannot spend the time with one patient, some patients will speak less, and some will bring the history of their family and tell us, my children are not taking care of us, and some start crying, in that situation we have to give them some time and other patients will be waiting for us* (N3a).

However, 6 months after starting the implementation, the pharmacist was transferred to another PHC. Soon after, the senior nurse was transferred and the doctor received a new assignment. These transfers were without replacement; therefore, at 9 months none of the QIs were implemented. The workflow became redundant, as a doctor was not available for consultations and prescriptions.

The involvement of ASHAs in the follow-up of patients was also not implemented 9 months into the implementation phase. The ASHAs were invited to the PHC for one meeting, where their roles in follow-up and lifestyle modification were discussed. The meeting was attended by most of the ASHAs (18 of 25), and they mentioned that they were already giving lifestyle advice but were not systematically following up with patients. However, further involvement did not materialize because the doctor was not available.

The QIs were implemented the least at PHC 1 and most at PHC 2. The implementation at PHC 3 started successfully, but when the team dissolved at 6 months, the implementation process came to an end.

### The local context of implementing QI

We analysed the data to identify locally relevant contextual factors at each of the PHCs. Major themes that emerged from this analysis related to the challenge of team-based care and the inability of staff to make changes within the strong hierarchal arrangement of the team. Team cohesion and motivation to implement the interventions also emerged as important themes.

#### Hierarchical arrangements and team-based care

The doctors are responsible for achieving the target indicators for their PHC. They have traditionally been placed at the apex of strong hierarchal arrangements among health professionals in India.

At PHC 1, the doctor did not often seek the opinion of his team in decision-making regarding the functioning of the PHC. The following quote from the interview with the lab technician illustrates how the doctor asserts authority and resists suggestions from staff.

*It is not possible to give any suggestions to our doctor. You know our doctor; no, he will not listen to anybody … he has repeatedly told in the past that he is the doctor and I am not the doctor, so I cannot suggest anything* (L1).

Even when the staff had suggestions or wanted to make changes in their areas of work, they were often not allowed to do so unless the doctor gave directions.

*Change means, whatever Sir says I will do like that, I cannot say, ‘I want to do this* (L1).

The doctor at PHC 2 was different in that he sought the opinions of his staff with regard to managerial decisions.

*All of us will discuss first, and if we feel this is correct and we can bring in the change we will go to Sir and tell him, he will bring the change* (N2b).

The team members, such as the lab technician and pharmacist, seemed to be able to make changes in their areas of work at the PHC.

*We can do, there is no restriction, we can do, we have to inform Sir, that we are doing this change and we can do* (N2b).

At PHC 3, the doctor did not seek the opinions of all staff equally regarding managerial decisions, as the opinion of the lab technician seemed to be valued more than the others. This may have been because the lab technician was well-qualified and had worked at the district hospital previously. Well-qualified staff flattened the hierarchical structure and enabled participation. The lab technician described how she was able to negotiate change with the doctor by virtue of being an ‘expert’ in her area of work:

*So me and madam [doctor] discussed and decided to have the ANC clinic on one day and NCD clinic on one day. We decided to have NCD clinic on Fridays every week and have started this* (L3).

We also observed that staff training and clear responsibilities empowered them to make decisions and participate in patient care. The nurse described the initial inhibition to make decisions independently and how this changed after roles were specified:

*Earlier, we used to say that Madam [doctor] has to write. Only if Madam writes we do sugar test, we were afraid that we have no permission to do. Now we have no fear if patients come, we do the tests* (N3a).

#### Team cohesion

The team members had specific roles related to their training, but all of the teams reported sharing each other’s work if possible and supporting each other, especially during absences due to leave. This was especially important, as we observed that the teams at all of the PHCs rarely had all members present on all days, due to leave, deputation for training, or supervisory visits from the district or state health authorities that demanded their attention.

The team at PHC 1 was the least cohesive of the three. They had no shared activities in which they participated. The pharmacist, in particular, was often not involved in the meetings or in causal discussions. However, they did help each other, especially when on leave, as illustrated by the lab technician:

*If I am on leave, and I am not available also, they will manage, if I give some work for them and ask them to do it on my behalf, and tell them that I am on leave, they will do it, like filling the details, everybody will support in our staff* (LT1).

We observed the most cohesion between team members at PHC 2. They would wait for each other during the lunch break to eat together:

*Our PHC is like a home, very little time we fight or else we are all one, especially during lunch time we are all one. I have worked in several other places, but here is the best, there they used to be on their own, but here everybody’s work we share and do* (LT2).

We also observed that tasks were redistributed by mutual agreement during the course of implementation when a member expressed an inability to complete the task due to a lack of time.

At PHC 3, the team was supportive of each other and helped each other with their work. We observed the team getting together during their break time on some days. However, the transfer of three members of the team limited our understanding of cohesion in this team.

*Here they are very supportive, if suppose we have to do FBS, but I come late, sisters here said no problem we will do it till you come* (LT3).

#### Motivation and perceived effect of the interventions

At all three PHCs, the staff perceived some changes due to the interventions, resulting in increased motivation among some of the staff. Interestingly, counselling had the greatest impact on personal motivation and satisfaction at the PHCs.

At PHC 1, the staff felt that the follow-up was better, as they now had records for each patient at the PHC.

*Very few patients were coming regularly for follow-up visits, they used to come casually, take tablets and go, but now they come correctly once in a month* (N1b).

At PHC 2, the effect of counselling was also a motivating factor, as illustrated by the lab technician, who was doing the counselling for patients:

*When we counsel a patient about the dos and don’ts for his diabetes, which they will not be knowing, and they listen to us, we will feel satisfied* (LT 2).

The lab technician at PHC 3 also expressed similar motivation and satisfaction after counselling patients:

*Just doing the tests, anybody can do that, but the counselling I have done and they have followed my instructions, and they have made use of it, that gives me a satisfaction that even I have contributed something* (LT 3).

The lab technicians at PHC 2 and PHC 3 had volunteered to complete this task because the nurse did not have time during the course of implementation. The lab technicians were accepted by patients and found it fulfilling to participate in this manner.

### Comparing the local context and the outcomes of implementation at the different PHCs

Although the PHCs are similar to respect to their roles, team structure and available infrastructure available, we found differences in team behaviour that may have affected the way the team came together to implement the interventions. The success of implementation at PHC 2 can be related to relatively more cohesive and participatory team dynamics. Another significant contextual factor at PHC 2 was the presence of the CC, which positively influenced the outcome of the intervention (Table 6).

**Table 6:** Comparison of implementation across PHCs

	PHC 1	PHC 2	PHC 3
Success of implementation	−	**+**	**±**
oWorkflow	−	+	±
oTask distribution	−	+	±
oRecord	+	+	±
oCHW F/U	±	±	±
Hierarchy within team	+	±	±
Team cohesion	−	+	+
Motivation	±	+	+
Main facilitators	Doctor’s interest	CC	Team interest
Main barriers	Time, patient load	Time, patient load	Team dispersed

−, indicates absence; ±, indicates sometimes present; +, indicates presence.

The CC was able to facilitate the workflow, follow-up with patients and serve as the focal point for the changes introduced:

*If one person is there to coordinate, it is helpful, or else just me to do everything will be difficult. We have care coordinator so he is the person and we are monitoring the cards* (D2).

From the analysis, it appears that a team leader (medical doctor) who is willing to include other members of the team in making decisions regarding functioning of the PHC and a cohesive team are important contextual ingredients for the implementation of QIs in primary care facilities. The presence of a CC who is able to facilitate the implementation of these changes can also be important.

## Discussion

We studied the implementation of QI interventions for NCDs at three public PHCs in rural South India and qualitatively assessed the implementation processes and outcomes. The three PHCs had different outcomes, as only one of the PHCs was able to make the changes and were implementing them at the end of 9 months. Comparing the local context at the three PHCs highlighted the role of hierarchical arrangements in the team and the lack of a team-based approach, which could be related to the different implementation outcomes. We also found that workflow management by a CC, team cohesion and motivation from feedback positively impacted the implementation process. These findings are not unique to the setting of rural publicly funded PHCs in India and have been reported in other primary care settings for persons with diabetes and hypertension (Khatib *et al.*, 2014; Rushforth *et al.*, 2016).

### Theoretical reflections

We found the CFIR framework to be a good guide for our study, as it helped define constructs, such as context and setting. We found that hierarchical structures influenced team-based care and impacted implementation related to the inner settings. We confirmed that centralizing or concentrating decision-making negatively impacts implementation. Similarly, team cohesion relates to the construct of social capital, defined as the quality and extent of relationships within the organization (Damschroder *et al.*, 2009). The CFIR further proposes that the bonding between members of the team influences implementation, and we found that this may have been a contributing factor to the relative success of implementation at PHC 2. The MUSIQ model focuses much more on interactions at the micro level during implementation processes (Kaplan *et al.*, 2012). The model hypothesizes that the internal ecosystem at each primary care facility is driven by the leader and directly impacts the implementation outcome. Leadership is an important theme in the MUSIQ model (Kaplan *et al.*, 2012) and all our findings (hierarchy, team cohesion, motivation, staff attrition) can be related to leadership at the three PHCs.

### Relevance of the results

Across different settings in primary care, leadership influences day to day functioning and impacts quality of care. Strong management and leadership competencies, such as motivating the team, have been identified as critical to enhancing health system performance (de Savingy and Taghreed, 2009; Yellappa *et al.*, 2016). Though leadership and management are theoretically distinct, they overlap in practice. Leadership is viewed as a process of enabling others to work, and management as a set of tasks, such as planning, budgeting and organizing (Daire *et al.*, 2014). It is this view of leadership that we found lacking, especially at PHC 1. Many studies have shown that team-based care, in which all members of the team play an integral role in providing patient care, is an effective tool in delivering high quality patient-centred care (Wen and Schulman, 2014; Wagner *et al.*, 2017; World Health Organization, 2018). However, strong enabling leadership is required to facilitate team-based care.

Leadership that is relational, combining a vision and sensitivity to the views of others, is considered to be more effective in bringing about QIs (Cleary *et al.*, 2018). Authoritative and hierarchical styles of leadership are associated with poor staff motivation, inability to work as a team and poor outcomes for patients (Jackson *et al.*, 2017). The WHO and the Alliance for Health Systems, in its flagship report, define participatory leadership as the ability to empower teams and engage communities to achieve better health outcomes (Report, 2016). This type of participatory leadership needs to be developed at all levels of the health system, but particularly at the primary care level and in the context of an LMIC. However, developing leaders that facilitate team-based care is especially challenging in countries like India, where doctors have traditionally been the sole providers of care.

Case studies of leadership in primary care in 12 countries have highlighted the role of training primary care leaders for effective leadership (Flahault *et al.*, 1986). In the Indian context, training and sensitization to relational aspects of leadership is an important first step. These skills are not part of the training in medical school; therefore, training courses may be one way to create awareness. Identifying role models and effective mentoring are other ways by which this capacity can be built over time. This also calls for studies of the complex interactions of leadership, context and system change in the Indian context using relevant methods (Gilson and Agyepong, 2018).

In this study, we experimented with task redistribution among the team members at primary care level. Redistribution of tasks or task shifting to members of the team other than the doctor is a viable, cost-effective solution to improving care for persons with chronic conditions in primary care in LMICs (Joshi *et al.*, 2014; Seidman and Atun, 2017; World Health Organization, 2018). In this study, the task of counselling was shifted from being the sole responsibility of the medical doctor to either the nurse or lab technician and the task of reminding patients to attend follow up at the PHC shifted to the ASHA in the community. While the task of counselling was well taken up by the nurse or lab technician, it proved difficult for ASHA workers to regularly follow up patients in their homes. Clearly defined roles and appropriate capacity building are recommended to enable task shifting (World Health Organization, 2007); in our study, we found this to be crucial in enabling the nurse or lab technician to counsel patients.

The positive feedback from patients regarding lifestyle advice received during counselling sessions motivated the nurse and lab technician to perform this additional task and is likely to have contributed to the relative success of this task shifting arrangement. A recent study of motivation and job satisfaction of health workers in Indian PHCs report that training sessions and the opportunity to use the acquired skills were important factors motivating health workers (Peters *et al.*, 2010).

The limited ability of ASHAs to follow up patients despite capacity building and clear role descriptions that we observed in this study, may at least partly be due to the lack of specific financial incentives. Indeed, ASHAs currently receive incentives from the Ministry of Health, for each of the health care services they provide, mainly related to maternal and child care. A recent study that evaluated the training of ASHAs for the control of hypertension also report that the lack of a financial incentive demotivates the ASHA from incorporating additional tasks into her routine (Abdel-All *et al.*, 2018).

Recent reviews pertaining to the context of LMIC, support task sharing and team-based care as a promising way forward to deliver chronic care at the primary level but also point to the necessity of supportive supervision (Anand *et al.*, 2019). Regulatory frameworks, enhanced job descriptions and a clear policy framework at the state level would be required to roll out task shifting at a wider scale (World Health Organization, 2007; Joshi *et al.*, 2018). In India the recent legislation introducing a new cadre of community health providers for primary care is a step in this direction (Ayog, 2016). Community health providers are envisaged to be persons associated with allopathic medical practice, such as nurses, that after the requisite training will be able to fulfil the role of medical doctors at the primary level of care. Other studies in similar settings have shown that non physician clinicians, appropriately trained, are as competent as physicians in providing primary care (Rao *et al.*, 2013).

We also found that the presence of a CC at the PHC facilitated the workflow and task redistribution. This highlights a possible need for additional staff that is not necessarily highly specialized, but that can play the role of a manager in coordinating patient care. In our study, the coordinator retrieved health records and guided the patient through the health facility. This role ensured a consistent point of contact for the patient at each visit to the health facility and facilitated the management of the patients’ health record. Both these activities contribute to continuity of patient care (Schwarz *et al.*, 2019), a crucial element in the care for persons with a chronic condition. While the availability of financial resources for an additional coordinating staff at the PHC may be challenging in India and other poorly-resourced settings, this role could be taken up by an existing, non-specialized staff at the PHC (Van Dillen and Hiddink, 2014). The policy option of introducing care coordination as an additional and specific task in delivering care for chronic conditions has not (yet) been researched thoroughly in the Indian context.

Our study was limited by the inclusion of only three PHCs where the doctors were willing to participate. Obviously, we therefore cannot generalize our findings to other Indian PHCs, and acknowledge that the varied motivation of doctors to engage in a QI initiative will impact implementation, even in similar settings. We are also aware that we were able to only assess some of the possible influencers in the implementation process. There may have been important factors in the external environment, such as the policies that shape the delivery of care at the PHC, which we were not able to capture, as the focus of the study was more on the inner settings of the individual PHCs. However, the in-depth understanding of the inner settings we gained in this study would have been difficult to achieve if we would have increased the number of PHCs or expanded the scope of the assessments. In addition, we have not related implementation success with the outcomes of the intervention, as we wanted to first understand the context and its role in the implementation process. The outcomes of the interventions will be reported in a subsequent paper.

Sixty per cent of mortality in LMICs can be attributed to poor quality of care, and the remaining to non-utilization of health services (Kruk *et al.*, 2018b). QI initiatives are greatly needed, especially at the primary care level in LMICs (Garcia-Elorrio and Schneider, 2012; Kruk *et al.*, 2018a). Investments in strengthening primary care such as infrastructure and equipment need to be accompanied by continuous QI efforts relevant to the local context. QI measures developed through participatory approaches, involving health care professionals, patients and communities are more likely to result in locally relevant and therefore sustainable QI (Nambiar *et al.*, 2017). Ghana presents one such example of continuous QI to support the implementation of a national maternal health programme that was scaled nationwide (Agyepong *et al.*, 2001; Twum-Danso *et al.*, 2012). It appears that a broad and deep stakeholder engagement, data-driven assessments, a health systems approach to QI, capacity building of leadership and immediate scaling of tested, locally relevant intervention packages as crucial elements for QI in their setting (Twum-Danso *et al.*, 2012). The findings of our study are in line with these conclusions and this seems to be a way forward to achieve QI in LMIC settings.

Delivery of high-quality care is the need of the hour, and requires continuous QI initiatives with attention to leadership, capacity building and genuine participation of all stakeholders.

## Conclusion

We found that the prevailing hierarchical relationships in primary care teams in India are a major barrier to team-based care and redistribution of clinical, organizational and managerial tasks at PHC level. This study draws attention to the need for building capacity and leadership to enable better implementation of public health programmes. Further research regarding the development of QI teams, testing QI intervention packages and studying organizational behaviour at primary care settings in India, is required to strengthen the delivery of primary health care for people with chronic NCDs.

## Funding

The research was supported by the Institute of Tropical Medicine, Antwerp, through a PhD scholarship to the first author. This article is part of the supplement ‘Innovations in Implementation Research in Low- and Middle-Income Countries’, a collaboration of the Alliance for Health Policy and Systems Research and Health Policy and Planning. The supplement and this article were produced with financial support from the Alliance for Health Policy and Systems Research. The Alliance is able to conduct its work thanks to the commitment and support from a variety of funders. These include our long-term core contributors from national governments and international institutions, as well as designated funding for specific projects within our current priorities. For the full list of Alliance donors, please visit: https://www.who.int/alliance-hpsr/partners/en/.

##  

Supplementary data are available at *Health Policy and Planning* online.

*Conflict of interest statement*. None declared.

*Ethical approval.* The study received ethical approval from the ethics committees of the Institute of Tropical Medicine, Antwerp (ref 1186/17), the University of Antwerp (ref 17/47/527) and the Institute of Public Health, Bengaluru (ref IEC-FR/02/2017). We also obtained permission from the State Ministry of Health and the Kolar District Health Office to conduct this research.

## Supplementary Material

czaa121_Supplementary_Data
