# Supplementary material for: Team-based primary health care for non-communicable diseases: complexities in South India
Source: Health Policy Plan. 2020 Nov 6;35(Suppl 2):ii22–34. doi: 10.1093/heapol/czaa121 (PMC7646724; doi:10.1093/heapol/czaa121)
Supplement: czaa121_Supplementary_Data [file czaa121_supplementary_data.zip › czaa121-suppl_data/Annex1_ final.docx]

# Primary Health Center, Kolar District

## Patient Health Record for treatment and follow-up of NCD Socio-demographic details

**Patient ID**

Date of registration:

Name of the patient:

S/o *I* D/o *I* W/o Age:

Gender:

Address: Area *I* out of area Door No.

Village Panchayal Taluk

Mobile number for contact:

**NCD diagnosis**

(Please circle all those that apply)

Diabetes mellitus type 2 Stroke

Hypertension Ischemic heart disease

Chronic kidney disease Hypercholesterolemia

## Risk factor assessment

(Please circle appropriate option) Screening for complications (annual)

| Family history  Any first degree relative had hypertension/diabetes/  heart attack/stroke? And Who?  Smoking | Yes / No  Relationship: Yes / No | Parameters | | Date | Findings |
| --- | --- | --- | --- | --- | --- |
|  |  |  | Eye a) Diabetic  retinopathy  b) Glaucoma  ECG findings |  |  |
| What do you smoke?  How long? (in years) | Beedi/ cigarette/ others |  | Albuminuria  Lipid profile LDL  1. HDL 2. Total cholesterol |  |  |
| How often? | / day |  |  |  |  |
| Alcohol  How long? (in years) | Yes/ No |  |  |  |  |
| How often?  Physical activity > 30 min | Daily *I* weekly once *I*  monthly once *I* occasionally  Yes/ No |  | HbA1c  Remarks / Problems identified |  |  |

# Patient Clinical Record

| Parameters | Visit 1 Date | Visit 2 Date | Visit 3 Date | Visit 4 Date | Visit 5 Date | Visit 6 Date |
| --- | --- | --- | --- | --- | --- | --- |
| Fasting blood glucose (mg/di) |  |  |  |  |  |  |
| Post prandial blood glucose (mg/di) |  |  |  |  |  |  |
| Blood pressure (mmHg) |  |  |  |  |  |  |
| BMI (as per chart) |  |  |  |  |  |  |
| Waist circumference  Signature of staff |  |  |  |  |  |  |

### Clinical Notes (please make a note of any abnormal ity or else write NAD)

| Foot examination  Local redness Pulses  Sensation |
| --- |
| General Examination |
| Medication |
|  |
|  |
| Notes |
| Follow-up dale Signature of doctor |

Counseling checklist

*Please check if advised*

| Diet  Low salt  Low fat  High intake of fruits and vegetables |  |
| --- | --- |
| Exercise >30 mins |  |
| Stop smoking and alcohol |  |
| Regular medication and follow-up |  |
| Lifestyle goal to achieve till next visit | |
| Remarks or problem identified |  |
| Signature of staff |  |
