# Supplementary material for: Team-based primary health care for non-communicable diseases: complexities in South India
Source: Health Policy Plan. 2020 Nov 6;35(Suppl 2):ii22–34. doi: 10.1093/heapol/czaa121 (PMC7646724; doi:10.1093/heapol/czaa121)
Supplement: czaa121_Supplementary_Data [file czaa121_supplementary_data.zip › czaa121-suppl_data/Annex2_final.docx]

**Interview guide for staff**

Regarding the organisation of care at PHC and the context

Objective of interview:

1. To understand the current processes of care at PHC

2. To understand if there has been any change

3. Understand staff perspectives of the changes

4. Understand why it did or didn’t work

5. Understand what else can be done to enhance implementation

**Topic 1 Personal story**

Q. Please tell us a little bit about yourself

When did you start working here? Where do you live? what did you study? Do you like working here? What are some things you like/don’t like?

**Topic 2 Services at the PHC**

Q. Can you describe what is currently happening (process) when a patient visits the health facility for diabetes/hypertension

Probes:

- Process - Where first and then
- Roles

Q. Was it always like this?

Q. What has changed?

Probes:

- Process
- Roles
- Recording

Q. Were there more changes that were supposed to be made? Were these implemented?

Q. What do you think about these changes?

Probes

- Role
- Capacity
- Usefulness/ benefit

Q. Why are these good and for who? Or are these not good, and for who?

Q. Why do you think it has not been implemented as planned

Probe:

- Team
- Support from doctor, or higher authorities
- Capacity
- Workload
- Flow and layout

Q. Why was it not possible to initiate FBS?

Q. What do you think are some reasons why we were not able to implement all that we wanted?

Q. Whose program do you think this initiative is?

Q. How else could it have been done?

Q. What can be done to improve implementation? How can we make this work better?

**Topic 3 Context**

Q. What do you perceive is the role of PHC in health care?

Q. What do you perceive is your role at this PHC?

Q. What is the structure? Are there hierarchies at this PHC?

Q. Why do you work at the PHC?

Q. Do you like working at the PHC? What are the nicest and worst parts of work?

Q. Do you feel you can share grievances or problems at work with each other?

Q. Do you feel you are heard and your suggestions considered?

Q. How are changes made to the work that you do?

Q. How do you perceive quality? What do you consider quality? Do you think you do quality work? How do you evaluate yourself?

Q. When/how can you say there is quality work done here?

Q. What do you think about the care given here?

- Drugs
- Testing facilities
- Doctor
- Counselling and support

Q. In what ways is quality assessed/evaluated at this PHCQ? Does the senior/superior/ doctor/lead speak to you all about quality of services?

Q. Do you feel you can make changes to your work, flow, or process?

Q. Have you made any changes?

Q. Is the community involved in decisions regarding service delivery? Should they be?

Q. How do you think this becomes part of the work you do?

Q. What do you think patients perceive of the services? Why do you think so? What may contribute to this? How can we improve this?
